# Optimizing Implant Placement Timing and Loading Protocols for Successful Functional and Esthetic Outcomes: A Narrative Literature Review

**DOI:** 10.3390/jcm14051442

**Published:** 2025-02-21

**Authors:** Panagiotis Rafail Peitsinis, Aikaterini Blouchou, Georgios S. Chatzopoulos, Ioannis D. Vouros

**Affiliations:** Department of Periodontology, School of Dental Medicine, Aristotle University of Thessaloniki, 54124 Thessaloniki, Greece; ppeitsini@dent.auth.gr (P.R.P.); amplouch@dent.auth.gr (A.B.); iovouros@dent.auth.gr (I.D.V.)

**Keywords:** delayed implant placement, dental implants, immediate implant placement, early implant placement, success, survival

## Abstract

**Objective**: This review article aims to analyze the existing relevant literature comparing the clinical outcomes and underlining the most common complications associated with immediate, early, and delayed dental implant placement in order to determine the most favorable timing for achieving optimal functional and esthetic results for the patient. **Methods**: A comprehensive review of the literature was conducted using PubMed-MEDLINE and Cochrane Library and a number of keywords, including “dental implant placement timing”, “immediate implant”, “early implant”, “delayed implant”, “clinical outcomes”, “complications”, and “implant success”, focusing on studies comparing immediate, early, and delayed implant placement. The primary outcome variable was implant survival rate, while secondary outcome variables included implant success rate, complications, and patient-reported outcomes. **Results**: A total of 9774 articles were identified. The articles included a variety of studies, including randomized controlled trials, prospective cohort studies, and retrospective studies. Immediate implant placement was associated with a high survival rate (93.8–100%), but also with an increased risk of complications, such as gingival recession and implant exposure. Early implant placement (4–8 weeks or 12–16 weeks after extraction) showed similar survival rates (95–100%) and fewer complications compared with immediate placement. Delayed implant placement (more than 4 months after extraction) was the most commonly used protocol and demonstrated high survival rates (92–100%) with predictable outcomes. Implant success rates varied depending on the criteria used, but all types of placements showed acceptable success rates (83.3–100%). The choice of loading protocol (immediate, early, or conventional) also influences treatment outcomes. **Conclusions**: The timing of dental implant placement and loading should be individualized based on patient-specific factors, such as bone and soft tissue conditions, medical history, esthetic considerations, and patient preferences. Immediate placement can be successful in ideal conditions but requires careful patient selection and surgical expertise. Early and delayed placement offer more predictable outcomes and are suitable for a wider range of patients.

## 1. Introduction

An endosteal implant is defined as an alloplastic material surgically inserted into a post-extraction alveolar ridge in order to replace one or multiple missing teeth. The innovative research project of Branemark and colleagues in the 1980s established the term osseointegration and laid the foundation for further investigation around the topic of implant placement and loading with a supportive prosthodontic restoration [1,2,3]. Nowadays, due to the wealth of clinical studies, systematic reviews, and meta-analyses, dental implants are widely used in the field of dentistry as a successful restorative approach following a tooth extraction, achieving unquestionably high survival and success rates in respect to aesthetics, function, and patient satisfaction [4,5,6,7].

Following tooth extraction, the alveolar socket undergoes dimensional changes during healing, with significant reductions in ridge width (up to 50%) and height occurring within the first year (6–12 months) in the molar and premolar region, primarily within the initial three months [8,9]. This remodeling process involves a series of stages: initial blood clot formation, inflammatory cell infiltration, granulation tissue replacement, woven bone deposition, and, finally, lamellar bone maturation [10,11,12]. These dynamic changes, influenced by factors such as cytokines, growth factors, and bone morphogenetic proteins (BMPs), impact the ideal timing of implant placement [8,13,14].

Premature placement, whether immediate or early, may lead to esthetic and functional compromise due to ongoing bone resorption and remodeling, particularly affecting the buccal/facial bone wall [8]. Therefore, a comprehensive assessment of each patient’s alveolar ridge morphology is crucial to determine the optimal timing for implant placement (immediate, early, or delayed) to achieve predictable and successful outcomes with respect to both esthetics and function.

**Figure 1:**
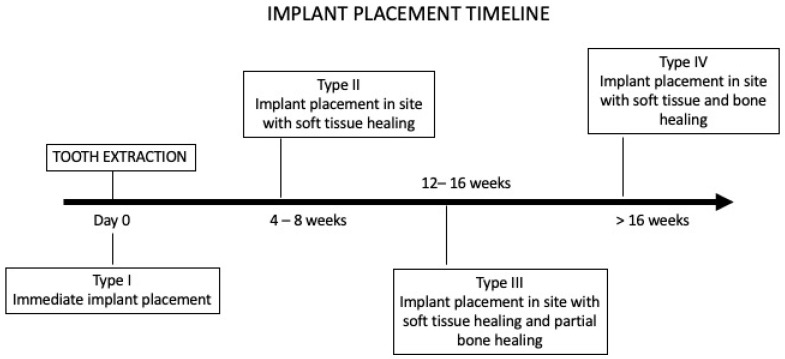
Implant placement timeline.

According to the literature, the timeline of implant placement includes four types of installation performed in different post-extraction stages [15,16]. Hämmerle and colleagues [17] introduced the classification of four categories (Type I–IV), which was later modified by Chen and Buser [18]. Figure 1 displays the implant placement timeline. Based on these guidelines, the protocol for implant installation has been established as:

- Τype I, or immediate implants, placed directly after tooth extraction;
- Type II, or early implants, with soft tissue healing, placed 4–8 weeks after tooth extraction;
- Type III, or early implants, with partial bone healing, placed 12–16 weeks after tooth extraction;
- Type IV, or delayed implants, with soft and hard tissue healing, placed 4 months after tooth extraction.

The timing of dental implant placement is critical to treatment success and survival, affecting healing, long-term stability, and the esthetic outcome. Immediate placement (Type I), when performed in ideal conditions, such as a lack of inflammatory factors, the presence of a thick gingival tissue phenotype, and intact facial bone walls (>1 mm), offers reduced treatment times and surgical interventions but requires meticulous patient selection and surgical expertise [17,19,20]. Factors including the thin tissue phenotype, a lack of keratinized mucosa for flap adaptation, and site morphology may negatively affect the clinical outcome and increase the risk of implant failure compared with delayed implants [21]. Early placement during initial healing (Type II) allows for soft tissue management and resolution of inflammation but may necessitate regenerative procedures due to ongoing bone remodeling [17,20]. The dynamic nature of the alveolar ridge during this period can lead to variations in the resorption rates of the socket walls, potentially influencing implant stability and final esthetic outcomes. This may necessitate the adjunctive use of regenerative techniques, such as guided bone regeneration (GBR), which involves the placement of bone grafts and barrier membranes to augment the deficient ridge. However, the implementation of GBR procedures can increase the treatment’s complexity, potentially requiring additional surgical interventions and increasing overall treatment costs [22].

Type III implant placement, classified as an early implant approach, exhibits greater predictability compared with earlier-stage interventions. This is attributed to the substantial completion of bone and soft tissue remodeling, simplifying surgical flap management [17]. The literature supports its efficacy in multi-rooted tooth regions, notably mandibular first molars [20]. However, limitations observed in Type II placements, such as extended treatment durations potentially impacting patient satisfaction, also apply to Type III. Consequently, while offering surgical advantages, Type III may necessitate careful consideration regarding patient expectations and treatment timelines. Type IV implant placement is often favored by surgeons following significant dimensional changes within the alveolar socket, contingent upon sufficient keratinized mucosa and primary implant stability. This approach demonstrates high success and survival rates [17,20]. However, limitations include an increased treatment duration, a potential need for adjunctive procedures, and variability in the remaining bone volume.

The following sections outline the indications for each implant placement protocol, highlighting the clinical conditions and patient factors that should be considered when making treatment decisions.

### 1.1. Indications for Immediate Implant Placement

The immediate placement and restoration of dental implants following tooth extraction necessitate meticulous diagnosis and treatment planning to achieve optimal outcomes, particularly in esthetically demanding regions like the anterior maxilla. While this approach offers advantages such as reduced surgical intervention, pain, and treatment duration, thereby increasing patient preference, its implementation should be restricted to cases presenting ideal clinical conditions [17,23]. Optimal immediate implant placement typically involves positioning the implant palatally or lingually within the alveolus to mitigate the risk of exposure during alveolar ridge resorption [24]. A critical prerequisite for successful osseointegration is the presence of an intact facial bony wall exceeding 1 mm in thickness, along with a thick gingival phenotype [20]. Atraumatic extraction techniques are advocated to preserve alveolar socket integrity and minimize soft tissue trauma [25]. Moreover, studies have demonstrated a correlation between a thin gingival phenotype and an increased risk of gingival recession after implant placement. This recession can result in compromised esthetics, including the exposure of the implant–abutment junction or even the implant fixture itself. Consequently, patients exhibiting a thick gingival phenotype are generally considered more favorable candidates for immediate implant placement due to the reduced likelihood of soft tissue complications [20]. Primary implant stability is paramount, necessitating placement at least 3 mm apical to the alveolar crest and achieving adequate insertion torque (25–40 N/cm) or an Implant Stability Quotient (ISQ) value of 70 or greater [26,27]. Flapless surgical approaches are often favored to reduce gingival recession and associated complications [20]. Patient selection criteria include the absence of uncontrolled systemic conditions that may compromise osseointegration. Factors such as heavy smoking, periodontal inflammation, and inadequate bone volume at the recipient site are considered contraindications and should be addressed prior to implant placement [15].

### 1.2. Indications for Early Implant Placement

Early implant placement, performed either 4–8 weeks or 12–16 weeks post-extraction, offers distinct advantages over immediate implantation [18]. This approach allows for partial bone healing and complete soft tissue maturation, facilitating optimal esthetic outcomes and simplifying the surgical procedure [18]. Osseointegration is enhanced by the establishment of primary stability, a critical determinant of implant success [28]. During this healing period, inflammatory factors are resolved, and new bone formation occurs apically within the alveolus. This facilitates precise implant bed preparation and optimal three-dimensional implant positioning. Unlike immediate implants, early implants can be successfully placed at sites with compromised facial bone walls, utilizing guided bone regeneration techniques to augment esthetics. The presence of partial bone healing and newly formed keratinized mucosa reduces the risk of mucogingival complications, particularly in areas with thin or deficient buccal bone. While immediate implants necessitate flapless surgery, early implant placement typically employs an open flap approach with a triangular flap design [20]. Although applicable to both Type II and Type III implant placement scenarios, Type III implants, placed 12–16 weeks post-extraction, are often preferred in multi-rooted tooth regions due to the extended healing period. Consequently, early implant placement presents a viable alternative to immediate implantation when conditions at the extraction site are suboptimal, offering enhanced predictability and improved esthetic outcomes.

### 1.3. Indications for Delayed Implant Placement

Delayed implant placement, occurring more than four months post-extraction, presents distinct advantages despite necessitating a longer treatment timeline and potentially multiple surgical interventions, particularly when GBR is indicated. This extended healing period allows for substantial alveolar ridge remodeling, resulting in predictable primary stability and facilitating optimal three-dimensional implant positioning and simplified surgical bed preparation [18]. Complete soft tissue maturation provides adequate keratinized mucosa, enabling meticulous flap elevation and minimizing the risk of esthetic complications [20]. Furthermore, delayed implantation allows for the completion of most bone remodeling processes [29,30]. However, GBR or sinus augmentation procedures may still be necessary to address a deficiency in bone volume, either at the time of implantation or immediately post-extraction for ridge preservation purposes.

Delayed implant placement is also indicated in patients who are not suitable candidates for immediate or early implant placement [20]. This includes young patients with skeletal immaturity, pregnant women, and individuals with uncontrolled systemic conditions that may impair healing. Additionally, the presence of significant local pathology, such as extensive apical lesions, necessitates delayed implantation to ensure adequate primary stability. In summary, while delayed implant placement requires a longer treatment duration, it offers enhanced predictability, simplified surgical execution, and a reduced risk of complications, particularly in complex clinical scenarios.

Despite extensive research on implant placement timing, several key gaps remain. There is a lack of long-term data on early implant placement, limiting our understanding of its efficacy and complications. Additionally, there is a need for more studies specifically evaluating implant success rates, encompassing functional, esthetic, and patient-reported outcomes, rather than focusing solely on survival. The literature also presents conflicting evidence on the relationship between placement timing and peri-implantitis, requiring further research to clarify this association. Furthermore, the lack of standardized reporting criteria hinders meaningful comparisons between studies, and there is a need for more cost-effectiveness analyses to guide treatment decisions. This review aims to evaluate the influence of implant placement timing (immediate, early, and delayed) on implant success rates, complication rates, patient-reported outcomes, and treatment timelines. Optimal dental implant treatment necessitates a comprehensive assessment of patient-specific factors, including anatomical limitations, age, medical history, and compliance with post-operative care [23]. We hypothesized that different implant placement timings (immediate, early, and delayed) will have varying impacts on implant success rates, complication rates, and patient-reported outcomes.

## 2. Methods

A comprehensive literature search was conducted using electronic databases including PubMed-MEDLINE and Cochrane Library. Keywords such as “dental implant placement timing”, “immediate implant”, “early implant”, “delayed implant”, “clinical outcomes”, “complications”, and “implant success” were employed. Inclusion criteria encompassed clinical studies, systematic reviews, and meta-analyses published in English up to January 2025 that directly compared immediate, early, and delayed implant placement protocols. Studies focusing on single-tooth and multiple-tooth replacements in both the maxilla and mandible were considered. Exclusion criteria included case reports, case series, animal studies, and in vitro studies.

The examined PICO question was:

Population: Patients requiring dental implant placement after tooth extraction.

Intervention: Different implant placement timings (immediate, early, and delayed).

Comparison: Comparison between the different implant placement timings.

Outcome: Implant survival rates, implant success rates, complications (e.g., peri-implantitis, bone loss, implant failure), esthetic outcomes, patient-reported outcome measures (PROMs), and treatment timelines.

Data extracted from the selected studies included implant survival rates, complications (e.g., peri-implantitis, bone loss, implant failure), esthetic outcomes, patient-reported outcome measures, and treatment timelines. The findings were synthesized narratively to provide a comprehensive overview of the clinical outcomes and complications associated with each implant placement timing protocol.

## 3. Results

A total of 9774 articles were identified. The articles included a variety of studies, including randomized controlled trials, prospective cohort studies, and retrospective studies.

### 3.1. Implant Loading

The timing of dental implant loading following implant placement (whether immediate, early, or delayed) is a critical factor influencing the long-term functional and esthetic success of the restoration. The International Team for Implantology (ITI) consensus statement (2018) defines implant loading protocols as follows [27]:

- Immediate loading: connection of dental implants with a prosthetic restoration in occlusion with the opposing arch within 1 week following tooth extraction (functional loading);
- Immediate restoration: connection of dental implants with a restoration within 1 week subsequent to implant placement and dental prostheses held out of occlusion (non-functional loading);
- Early loading: connection of dental implants with a prosthetic restoration in occlusion with the opposing arch between 1 week and 2 months after implant placement;
- Conventional loading: dental implants undergo a healing period of more than 2 months after tooth extraction and implant placement and during that period they remain not connected to the restoration.

To determine the most suitable implant placement and loading strategy for each patient, clinicians must consider various factors that can influence treatment outcomes. According to Morton and colleagues [31], these factors include the patient’s overall health and adherence to treatment, the initial stability of the implant, and the potential need for bone augmentation. Generally, good patient health, sufficient bone quality and quantity, an absence of infection, and primary implant stability are crucial for successful implant placement, particularly when immediate or early loading is desired. Immediate implant placement with immediate loading is a complex procedure that should only be performed by experienced clinicians, as it requires specific expertise and careful management of the forces on the implant during healing. Early implant placement is often a suitable option, but it may necessitate bone augmentation procedures and typically involves conventional loading. Delayed implant placement, where the implant is placed after a longer healing period, is often combined with early or conventional loading, as these protocols are well-established and considered routine. Ultimately, the choice of implant placement and loading strategy should be tailored to the individual patient, taking into account their specific needs and circumstances to achieve the best possible treatment outcome.

### 3.2. The Enigma of Implant Success: Unraveling the Complexities

A review of the past 20 years of research reveals consistently high survival rates (87.5–100%) for dental implants, regardless of the timing of placement (Table 1). Table 1 presents a summary of studies that evaluated implant survival rates based on the timing of placement (immediate, early, or delayed). The table includes information on the study design, purpose, number of patients and implants, age range, survival rate, and follow-up period. This allows for a comparison of survival outcomes across different implant placement protocols and highlights the reported survival rates associated with each timing type. While immediate implants (Type I) demonstrate acceptable survival rates, they are highly technique sensitive and rely on ideal conditions. Early implantation (Types II and III), often necessitating guided bone regeneration (GBR), exhibits excellent survival rates in the majority of studies. However, limited long-term data restrict definitive conclusions regarding its efficacy. Delayed implants (Type IV) offer a balance between immediate and early approaches, demonstrating predictable results.

While implant survival merely indicates the presence of the implant or restoration at a follow-up examination, implant success encompasses a broader range of criteria. Implant success is a multifactorial concept, influenced by systemic health, anatomical factors, patient habits, surgical techniques, and implant materials [58]. Key clinical parameters assessed during follow-up include the Pink Esthetic Score (PES), which evaluates soft tissue health and esthetics, and marginal bone loss (MBL), a critical prognostic factor [32,33]. Galindo-Moreno et al. [59] suggest that an MBL of less than 0.5 mm at 6 months post-placement can serve as an objective criterion for implant success. Plaque control and gingival health, as measured by the plaque index (PI) and gingival index (GI), are also essential for long-term success. Furthermore, patient satisfaction is a crucial component of successful implant outcomes, reflecting the subjective experience of the individual. The studies analyzing and comparing the success rates between the different implant placement types are shown in Table 2. Table 2 focuses on studies that analyzed implant success rates in addition to survival rates. Implant success encompasses a broader range of criteria beyond survival, including factors such as bone loss, prosthetic complications, and patient satisfaction. The table provides details on the study design, purpose, number of patients and implants, age range, success rate, and follow-up period for each study. This allows for a comparison of success rates across different implant placement timings and provides a more comprehensive assessment of treatment outcomes. This comprehensive review included 39 studies that investigated various aspects of implant placement timing and their impact on treatment outcomes.

**Figure 2:**
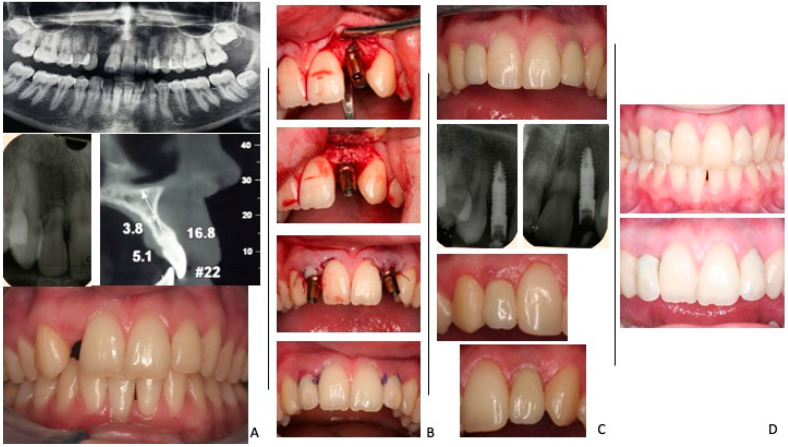
(**A**) Preoperative radiographic and clinical presentation of a patient with failing tooth #22 indicated for extraction and implant placement, and a congenitally missing #12, (**B**) Intraoperative view of immediate implant placement at the site of tooth #22 extraction, with simultaneous bone augmentation. Delayed implant placement at the site of tooth #12 extraction. (**C**) Final implant-supported restorations in place on both #12 and #22. (**D**) Ten-year follow-up clinical photograph demonstrating healthy peri-implant tissues.

Our review of the recent literature revealed a limited number of studies focusing on implant success rates, highlighting the need for long-term evaluations to assess outcomes accurately. Type I implants, when tested for success criteria, demonstrated the lowest success rates (66.7–100%). Early implant placement (Types II and III) showed higher success rates (79.16%), although research on this approach is limited. Delayed implants (Type IV), commonly used in practice, exhibited success rates ranging from 83.3 to 100%, generally demonstrating superior outcomes compared with immediate and early placement. The long-term outcomes of immediate and delayed implant placement are shown in a case with a 10-year follow up in Figure 2.

While immediate implants generally demonstrate acceptable survival rates, it is crucial to acknowledge the influence of the implant placement site on these outcomes. A study by Ramalingam et al. (2015) [71] specifically evaluated immediate implant survival based on the size and site of placement, finding variations in survival rates depending on the location. Their research suggests that immediate implants placed in the anterior maxilla may have a higher risk of failure compared with those placed in other regions. This highlights the importance of considering the specific implant site when evaluating the potential for success with immediate placement protocols. Factors such as bone quality, soft tissue thickness, and esthetic demands vary across different sites and can significantly influence the survival and overall outcome of immediate implants.

### 3.3. Maximizing Implant Success: A Focus on Surface Characteristics and Placement Timing

Implant surface characteristics play a crucial role in osseointegration, initial stability, and long-term success across all implant placement timings. The macro-design of the implant, including the thread shape and configuration, significantly influences primary stability by maximizing bone-to-implant contact and facilitating new bone formation [72]. Implants with a smaller pitch, and therefore a greater number of threads per unit length, exhibit enhanced stability and improved load distribution, particularly those with aggressive thread designs [72,73].

Surface roughness is a critical factor in the micro-design of dental implants, directly impacting osseointegration [74]. Increased roughness has been shown to accelerate osseointegration and improve success rates [75]. Surface modifications, such as sandblasting and acid-etching (SLA), enhance roughness and promote osteoblast activity, leading to faster healing and greater stability [74,76]. Bioactive coatings, such as hydroxyapatite and extracellular matrix proteins, further enhance osseointegration, while antimicrobial coatings, like tetracycline and chitosan, can minimize the risk of infection [77].

The importance of implant design and surface is amplified in immediate and early placement protocols, where the risk of complications is higher compared with delayed placement. Roughened implant surfaces have demonstrated superior outcomes in these scenarios, facilitating osseointegration in a less predictable environment [74].

### 3.4. Peri-Implantitis and Implant Placement Timing

Peri-implantitis, a pathological condition affecting the hard and soft tissues surrounding dental implants, is a significant cause of implant failure. It is characterized by inflammation in the peri-implant connective tissue and loss of supporting bone, distinguishing it from peri-implant mucositis [78]. The recent literature presents conflicting evidence regarding the influence of implant placement timing on peri-implantitis rates. Parvini et al. [79] found no significant difference in peri-implantitis rates between immediate and delayed implant placement but reported a high prevalence of peri-implant mucositis in over 30% of immediately placed implants. They also identified a lack of keratinized mucosa (less than 2 mm) as a risk factor for gingival recession and peri-implantitis. Conversely, Lanza et al. [80] found no conclusive evidence that any specific placement timing protocol (immediate, early, or delayed) conferred superior peri-implant health. However, they highlighted a history of periodontitis, particularly in combination with smoking, as a significant risk factor for peri-implantitis across all placement timings. Similarly, Bassir et al. [81] reported no significant differences in peri-implant probe depths, soft tissue levels, or overall peri-implant health between immediate and early implant placement.

### 3.5. The Role of Patient Expectations and Satisfaction in Implant Treatment Planning

In addition to achieving successful clinical outcomes, clinicians should prioritize fulfilling patient expectations regarding the final restorative results, as measured by patient-reported outcome measures (PROMs). Patients seeking dental implant treatment aim to restore edentulous areas and improve their quality of life. Minimizing discomfort during surgery and post-operative morbidity, such as pain and swelling, is crucial for patient satisfaction. Furthermore, shorter treatment times and reduced reliance on temporary restorations are generally preferred, along with optimal esthetics and functionality of the final prosthesis. While the literature indicates no strong correlation between implant placement timing and overall patient satisfaction [82,83,84], a statistically significant preference for immediate loading has been observed in edentulous patients receiving full-arch implant-supported restorations, likely due to the aforementioned advantages [82].

## 4. Discussion

This narrative review analyzed 39 studies on implant placement timing and loading protocols, examining their impact on implant success, complications, and patient-reported outcomes. The review also explored the influence of implant surface characteristics on osseointegration and long-term success. Additionally, it discussed the risk of peri-implantitis and the importance of patient expectations in implant treatment planning. The findings reveal that immediate implants have the highest risk of complications, while delayed implants offer the most predictable outcomes. Early implants show similar success rates to immediate implants with fewer complications. The choice of loading protocol also significantly influences treatment outcomes. Implant surface characteristics, such as macro-design and roughness, play a crucial role in osseointegration and long-term success. The literature presents conflicting evidence on the relationship between implant placement timing and peri-implantitis risk. Patient expectations and satisfaction are essential factors to consider in implant treatment planning.

### 4.1. The Role of Technology in Modern Implant Dentistry

Advancements in technology have revolutionized the field of implant dentistry, with computer-assisted or template-guided implant surgery playing a significant role in enhancing the precision and predictability of implant surgery, especially when it pertains to immediate and early implant placement. The integration of Cone Beam Computed Tomography (CBCT), as a 3D radiographic method in the preoperative evaluation for each patient, assists the clinician in designing the ideal treatment plan with precision, considering critical anatomical structures (inferior alveolar nerve, mental nerve, maxillary sinus floor perforation, etc.) [85]. Two types of guided implant surgery protocols—static and dynamic—are described in the literature [86]. A major benefit of these techniques is flapless surgery that tends to decrease some complications like pain, discomfort, swelling, and trauma [87], but also is perfectly aligned with immediate implant placement.

During the static protocol (s-CAIS), evidence from the CBCT is collocated with data for hard and soft tissues given by an intraoral scan. Subsequently, the use of advanced software is essential to import and superimpose the standard tesselation language (STL) data (from the intraoral scan) and the digital imaging and communications in medicine (DICOM) data from CBCT in order to digitally plan the most ideal insertion site for the implant in accordance with the future prosthetic restoration and anatomy restrictions, printing a surgical drill template that guides the clinician during the placement [88]. By extension, this surgical drill guide with sleeve guides applied is fabricated with CAD/CAM technology and can be supported by either the teeth, mucosa, or bone or specially supported with mini-implants or pins depending on each patient’s mouth characteristics [89]. Static guided surgery can be fully guided (FG), half-guided, and freehand (FH) [90]. However, the static protocol displays several disadvantages, such as an increased cost compared with the freehand procedure, insufficient water cooling during surgery that can cause tissue heating, bone resorption, and non-osseointegration of the implant, an inability to change the surgical plan intraoperatively, and difficulty with placing an implant in posterior regions due to the limited mouth opening [88,91].

Dynamic navigation in implant surgery (d-CAIS) offers real-time adjustments during the procedure, enhancing precision. While this technology offers benefits such as improved visibility and efficient cooling, it requires expensive equipment and a significant learning curve for clinicians [91]. Several studies measuring angular, cervical, apical, and depth deviation suggest that the accuracy of the static protocol and dynamic navigation are comparable, and both are superior to the freehand procedure [92,93,94]. A considerable factor affecting the success of both the static method and dynamic navigation is human error, such as hand tremors, fatigue, or inexperience, and this cannot be avoided. As technology continues to evolve, robotic computer-assisted implant surgery (r-CAIS) seems to be a very promising approach, providing even further precision and better results, even in conditions that have remained challenging throughout the ages, such as a successful immediate implantation in the anterior esthetic region [91,95].

### 4.2. Limitations

This review has certain limitations that should be considered. First, the included studies varied in their methodologies, sample sizes, and follow-up periods, which may introduce heterogeneity and limit our ability to draw definitive conclusions. Second, there was a lack of long-term data for certain implant placement timings, particularly for early implant placement, making it difficult to assess the long-term efficacy and complications of this approach. Third, the potential for bias in the included studies cannot be excluded, as we did not perform a formal risk of bias assessment. Finally, our search strategy was limited to PubMed-MEDLINE and Cochrane Library, and only included articles published in English, potentially missing relevant studies in other databases or languages. Future research should prioritize long-term studies with standardized reporting criteria to address these limitations and provide more robust evidence for clinical decision-making.

### 4.3. Future Research

Future research should prioritize long-term studies evaluating implant success and survival rates across various placement timings, utilizing standardized reporting criteria to ensure meaningful comparisons. These studies should incorporate patient-reported outcome measures (PROMs) to assess satisfaction, quality of life, and functional outcomes. Additionally, further investigation is needed to identify specific risk factors for peri-implantitis and develop effective prevention strategies, including the role of genetic factors, systemic conditions, and oral hygiene practices.

Furthermore, research should focus on optimizing implant placement and loading protocols, particularly for immediate and early placement, by refining surgical techniques, implant design, and surface characteristics. Evaluating the effectiveness of new technologies, such as computer-guided surgery and robotic surgery, in improving implant outcomes and patient satisfaction is also crucial. Finally, studies comparing the cost-effectiveness of different implant treatment modalities and investigating implant outcomes in specific patient populations, such as those with systemic diseases or limited bone availability, will further enhance treatment strategies and patient care.

## 5. Conclusions

In conclusion, the optimal timing for dental implant placement should be determined on a patient-specific basis, considering factors such as bone and soft tissue conditions, medical history, esthetic demands, and patient preferences. Immediate implant placement can be a viable option in ideal clinical scenarios with adequate bone and soft tissue support, offering potential benefits such as a reduced treatment time and preservation of the alveolar ridge. However, careful patient selection and meticulous surgical technique are crucial in order to minimize complications and achieve predictable outcomes.

Early implant placement, with or without bone grafting, can be considered when immediate placement is not feasible due to factors such as infection or an inadequate bone volume. This approach allows for some healing to occur while potentially minimizing alveolar ridge resorption. Delayed implant placement remains a reliable option, particularly in cases with a compromised bone volume or anatomical limitations, as it allows for complete healing and predictable implant positioning.

Ongoing research and technological advancements, such as computer-guided surgery and improved implant surfaces, continue to refine implant protocols and enhance outcomes. As the field of implant dentistry evolves, a deeper understanding of the biological and biomechanical factors influencing osseointegration will enable clinicians to provide increasingly predictable and successful implant therapies tailored to individual patient needs.

## Figures and Tables

**Table 1:** Studies analyzing and comparing survival rates between the different implant placement types (Type I, II, III, and IV).

Study	Year of Publication	Study Design	Purpose of the Study	Timeline of Implant Placement	No of Patients	Age Range (Years)	No of Implants	Survival Rate (%)	Follow-Up Period (Months)
Tortamano et al. [32]	2010	Prospective study	Evaluate survival rates in immediate implant placement and restoration in esthetic zone	Type I	12	-	12	100	18
Chatzopoulos et al. [33]	2022	Retrospective study	Estimate survival rates and factors affecting the outcome of immediate placement versus delayed implant placement	Type I vs. Type IV	4519	18–93 (60.27 ± 13.11) Group 1: 59.89 ± 14.10 Group 2: 60.30 ± 13.05	4519	98.5 vs. 98.9	32.27 (mean follow-up)
Raes et al. [34]	2018	Prospective cohort study	Evaluate clinical outcome after 8–10 years of immediate implants versus delayed	Type I vs. Type IV	39	(mean age 45 years for Type I, mean age 40 years for Type IV)	39 (16 Type I, 23 Type IV)	93.8 vs. 100	96
Wipawin et al. [35]	2024	Prospective study	Evaluate survival rates in immediate implant placement in the posterior region	Type I	19	33–76 (mean 56 ± 15.03)	25	96	57
Soydan et al. [36]	2013	Retrospective study	Evaluate success and survival rates between immediate and early implant placement	Type I vs. Type II	36	34–71 (mean 51.2)	50 (26 Type I, 24 Type IV)	96.16 vs. 100	51.6 vs. 61.9
Hamouda et al. [37]	2013	Preliminary study	Assessment of a modified insertion technique for immediate implant placement in mandibular molar sites	Type I	20	20–56 (mean 32.2)	20	95	18
Chappuis et al. [38]	2018	Prospective case series	Evaluate implants with simultaneous GBR after 10 years of function—early implant placement	Type II	20	35–71 (median age 53)	20	100	126
Buser et al. [39]	2013	Prospective cross-sectional study	Analyze the concept of early implant placement with simultaneous contour augmentation	Type II	41	38.8	41	100	60–108 (average follow-up of 84 months)
Thoma et al. [40]	2024	Randomized Controlled Trial	Early implant placement versus ridge preservation and delayed implant placement	Type II vs. Type IV	52	(mean 63.9 for Type II, 61.4 for Type IV)	52 (26 Type II, 26 Type IV)	100 vs. 92	9
Fugazzotto et al. [41]	2008	Retrospective analysis	Evaluate survival rates of immediate implants at the time of maxillary molar extraction	Type I	386	20–80+	391	99.5	75
Cucchi et al. [42]	2017	Randomized controlled trial	Estimate outcome of tapered, double-lead thread single implants in fresh extraction sockets versus healed sites of posterior sites	Type I vs. Type IV	92	20–79 (mean 51.0 ± 9.5)	97	95.9 vs. 100	24.5 ± 8.9 (test group or Type I) 24.3 ± 9.7 (control group or Type IV)
Belser et al. [43]	2009	Retrospective study	Evaluate outcome of early implant placement in the maxillary anterior region	Type II	45	17–81 (mean 39.9)	45	100	24–48
Chen et al. [44]	2017	Prospective study	Immediate implant placement simultaneously with sinus augmentation	Type I	37	Group 1 (29–46), Group 2 (25–49), Group 3 (35–59)	37	100	12
Malchiodi et al. [45]	2016	Randomized controlled trial	Primary and secondary stability of immediate versus early implants	Type I vs. Type II–III	40	54 (median) for immediate placement 51 (median) for early placement	40 (20 implants for Type I and 20 for Type II–III)	100 vs. 100	12
Fugazzoto et al. [46]	2009	Prospective study	Estimate the survival rate of immediate implant placement in mandibular molar regions	Type I	320	26–81	341	99.1	72 (mean 30.8)
Chen et al. [47]	2020	Prospective case series study	Evaluate alveolar ridge preservation and early implant placement at maxillary central incisor sites	Type III	10	21.8–71.7 (mean 42.9 ± 19.37 years)	10	100	12
Arora et al. [48]	2018	Prospective study	Evaluate clinical outcome of Type 1 and 2 placement protocols in single-tooth gaps	Type I vs. Type II	30	55.7 ± 13.3 for Type I placement 49.2 ± 13.8 for Type II placement	30 (15 placed immediately, 15 early placed)	100 vs. 100	12
Amato et al. [49]	2018	Retrospective clinical study	Investigate survival rate of immediate implant placement in molar extraction sockets	Type I	102	-	107	99.06	12–72 (mean 36)
Eghbali et al. [50]	2012	Cross-sectional study	Evaluate the survival rate for single implant treatment in healing versus healed sites of anterior maxilla	Type II vs. Type IV	48 (only 44 patient records available	23–76 (mean age 52)	49 out of 53 (22 Type II, 27 Type IV)	95 vs. 93	30
Botticelli et al. [51]	2008	Prospective study	Estimate the 5-year clinical outcome of immediate implants	Type I	18	21–81 (mean 49.1)	21	100	60
Cosyn J et al. [52]	2011	Prospective study	Assess the overall outcome of immediate implant placement in anterior maxilla	Type I	30	24–76 (mean 54)	30	96	36
Hirani et al. [53]	2023	Retrospective study	Survival of immediate implants replacing traumatized teeth in anterior maxilla	Type I	60	21–61 (mean 34.5)	70	95.7	36
Hakobyan et al. [54]	2020	Comparative analysis	Evaluate effectiveness of immediate versus delayed implant placement	Type I vs. Type IV	52 (28 Type I, 24 Type IV)	26–43	64	97.8 vs. 98.1	60
Carini et al. [55]	2014	Clinical Trial	Estimate clinical outcome of immediate loading with 2 different placement protocols (immediate vs. early placement)	Type I vs. Type II	10	(mean age 47.4)	15	87.5 vs. 100	12
Annibali et al. [56]	2011	Retrospective case series	Review the clinical outcome of immediate, early, and delayed single-tooth implant placement in mandibular or maxillary 1st molar sites	Type I (group 1) vs. Type II (group 2) vs. Type IV (group 3)	47 (19 patients at Group 1, 11 patients at Group 2, 17 patients at Group 3)	(mean) Group 1: 38.31 ± 12.08 Group 2: 41.3 ± 11.8 Group 3: 42.41 ± 14.3	53 (20 Type I, 12 Type II, 21 Type IV)	100 vs. 100 vs. 100	(mean) Group 1: 38.84 ± 16.14 Group 2: 32.91 ± 18.49 Group 3: 42.66 ± 12.41
Schropp et al. [57]	2014	Randomized controlled trial	Present 10-year clinical and radiographic data on single-tooth implants (Type I, III, and IV implant placement)	Type I vs. Type III vs. Type IV	63 (47 attended all control visits)	55.2 (mean)	63 (47 at all control visits: 14 Type I, 17 Type III, 16 Type IV)	91 vs. 95 vs. 100	120

**Table 2:** Studies analyzing and comparing success rates between the different implant placement types (Type I, II, III, and IV).

Study	Year of Publication	Study Design	Purpose of the Study	Timeline of Implant Placement	No of Patients	Age Range (Years)	No of Implants	Success Rate (%)	Follow-Up (Months)
Annibali et al. [56]	2011	Retrospective case series	Review the clinical outcome of immediate, early, and delayed single-tooth implant placement in mandibular or maxillary 1st molar sites	Type I (group 1) vs. Type II (group 2) vs. Type IV (group 3)	47 (19 patients at Group 1, 11 patients at Group 2, 17 patients at Group 3)	(mean) Group 1: 38.31 ± 12.08 Group 2: 41.3 ± 11.8 Group 3: 42.41 ± 14.3	53 (20 Type I, 12 Type II, 21 Type IV)	95 vs. 91.7 vs. 100	(mean) Group 1: 38.84 ± 16.14 Group 2: 32.91 ± 18.49 Group 3: 42.66 ± 12.41
Simsek et al. [60]	2003	Retrospective study	Evaluate success rates of immediate and delayed placement of implants	Type I vs. Type IV	80	17–62 (median 43 years)	310 (76 Type I, 234 Type IV)	93.4 vs. 95.7	24
Soydan et al. [36]	2013	Retrospective study	Evaluate success and survival rates between immediate and early implant placement	Type I vs. Type II	36	34–71 (mean 51.2)	50 (26 Type I, 24 Type IV)	76.92 vs. 79.16	51.6 vs. 61.9
Vandeweghe et al. [61]	2012	Clinical Trial	Study on a novo wide-body implant for posterior regions with 2 different protocols, immediate and delayed	Type I vs. Type IV	75	25–82 (mean 58)	93 (69 Type I, 24 Type IV)	86.2 vs. 93.5	14
Koh et al. [62]	2011	Randomized controlled trial	Identify factors that influence immediate implant placement	Type I	20	55.5 ± 3.3	20	95.8	4
Malchiodi et al. [45]	2016	Randomized controlled trial	Primary and secondary stability of immediate versus early implants	Type I vs. Type II–III	40	54 (median) for immediate placement 51 (median) for early placement	40 (20 implants for Type I and 20 for Type II–III)	100 vs. 100	12
E. H. van der Meij et al. [63]	2005	Retrospective study	Evaluate two endosteal implants in each patient and iliac crest onlay grafts in an atrophic mandible	Type I	17	37–69 (mean 56)	34	88.2	51.6
Eghbali et al. [50]	2012	Retrospective cross-sectional study	Effectiveness of single-implant treatment in healing versus healed sites of anterior maxilla	Type II vs. Type IV	44	23–76 (mean 52)	49 (22 Type II, 27 Type IV)	81 vs. 90	3
Belser et al. [43]	2009	Retrospective cross-sectional study	Assessment of the concept of early implant placement for use in esthetically sensitive anterior maxilla	Type II–III	45	17–81 (mean 39.9)	45	100	31.44 (24–48)
Heinenmann et al. [64]	2013	Controlled clinical trial	Estimate bone level changes in immediate and delayed implants using a platform-switched design	Type I vs. Type IV	58 (TG or Type 1: 35, CG or Type IV: 23)	56.4 ± 12.2 for TG and 61.8 ± 12.3 for CG	136	100 vs. 100	12
Zuiderveld et al. [65]	2017	Randomized Clinical Trial	Assess the outcome of connective tissue grafting on the mid-facial mucosa level of immediately placed single-tooth implants	Type I	60 (Group I or TG: 30 Grafted with connective tissue, Group 2 or CG: Without graft	CG: 47.8 ± 16.5 TG: 45.5 ± 15.5	60	96.7	12
Slagter et al. [66]	2016	Randomized Controlled Trial	Evaluate immediate single-tooth implant placement in bony defects in the esthetic zone versus delayed placement	Type I vs. Type IV	40	18–72 overall (For Type Ι: 18–63 mean 43.7 ± 13.9, for Type IV: 20–72 mean 48.6 ± 16.4)	40 (20 Type I, 20 Type IV)	100 vs. 100	12
De Bruyn et al. [67]	2012	Prospective multicenter clinical study	Comparing bone and soft tissue changes after immediate implant loading in healed ridges and extraction sockets	Type I vs. Type IV	113 55 at Group 1 (Type I) 58 at Group 2 (Type IV)	G1: 45 (mean) G2: 42 (mean)	113 (55 Type I, 58 Type IV)	87 vs. 92	36
Chappuis et al. [38]	2018	Prospective case series study	Examination of the effectiveness of early implant placement with simultaneous contour augmentation through guided bone regeneration—10 year follow-up	Type II–III	20	35–71 (median 53)	20	95	120
Atieh et al. [68]	2013	Controlled clinical trial	Estimate the success rate of immediate single-implant restorations in mandibular molar extraction sockets versus delayed placement	Type I vs. Type IV	24	51.5 for Type I 54.6 for Type IV	24 (12 Type I, 12 Type IV)	66.7 vs. 83.3	12
Yoshino et al. [69]	2014	Randomized controlled prospective study	Evaluate implant success and peri-implant tissue response following single immediate implant placement	Type I	20	27–87 (mean 52.6)	20	100	12
Schiegnitz et al. [70]	2024	Retrospective multicenter clinical study	Compare clinical and radiological outcomes of novel, fully tapered tissue-level implants in immediate vs. delayed placement protocols	Type I vs. Type IV	165 (50 patients Type I, 115 patients Type IV)	18–86 (24–84 for Type I with mean 59.9 ± 17, 18–86 for Type IV with mean 59.2 ± 15)	318 (68 Type I, 250 Type IV)	98.5 vs. 97.6	12

## Data Availability

No applicable.
